# Zwei Jahre im Krisenmodus — die kommunalen Finanzen bleiben trotzdem stabil

**DOI:** 10.1007/s10273-022-3255-8

**Published:** 2022-09-12

**Authors:** Florian Boettcher, Ronny Freier

**Affiliations:** 1 Ref. 304 (Kommunale Finanzen), Ministerium für Heimat, Kommunales, Bau und Digitalisierung, Jürgensplatz 1, 40219 Düsseldorf, Deutschland; 2 Öffentliche Finanzwirtschaft, Technische Hochschule Wildau, Hochschulring 1, 15745 Wildau, Deutschland

**Keywords:** H11, H71, H72

## Abstract

Die kommunalen Finanzen stehen seit Beginn der Coronakrise unter besonderer Beobachtung. Die deutsche Politik hat zurecht erkannt, dass bei der Bekämpfung der Krise eine handlungsfähige Kommunalpolitik zwingend notwendig ist. Tatsächlich haben die kommunalen Haushalte sowohl 2020 als auch 2021 mit Überschüssen abgeschlossen. Wie dieser Beitrag zeigt, sind die Gründe dafür sehr unterschiedlich. Die auskömmliche Finanzierung der Kommunen ist Garant für die effiziente Bewältigung der Krise vor Ort und die Rekordzahlen bei den kommunalen Investitionen sind der richtige Impuls in der Wirtschaftskrise.

## Abstract (en)

Federal and state policies have massively supported the German municipalities during the coronavirus crisis. As a result, municipal budgets in Germany have had a small surplus in 2020 and in 2021. The precise reasons for these surpluses are quite different in both years. In 2020, the additional expenditure as well as the enormous decrease in municipal tax revenue could only be overcome by large spending policies at federal and state level. In 2021, in contrast, the municipalities were back to collecting significant taxes themselves and were mostly self-sustaining. Still, municipal finances in 2021 profited from permanent policy changes regarding the trade tax levy (Gewerbesteuerumlage) and the federal government’s increased co-funding of accommodation costs.

Die deutschen Kommunen stehen bei der Bekämpfung der Coronapandemie in der ersten Reihe. Hier werden die Gesundheitsämter und der Schulbetrieb organisiert sowie Krankenhäuser betrieben. Um diese krisenbedingten Aufgaben stemmen zu können, sind schon früh in der Krise die kommunalen Finanzen in den Blick von Wissenschaft (Freier und Geißler, 2020) und Politik[1] gekommen. Zu Beginn der Krise im März 2020 stand zu befürchten, dass die Kommunen durch Mehrausgaben und Steuereinbrüche in großer Zahl Haushaltssperren aussprechen müssen. Solche haushaltsrechtlichen Schritte hätten nicht nur eine erschwerte Krisenbewältigung vor Ort bedeutet. Es wäre auch zu befürchten gewesen, dass geplante Investitionen auf Eis gelegt werden, was für eine schnelle wirtschaftliche Erholung sehr kontraproduktiv gewesen wäre. Der Bund und die Länder haben die drohende Notlage schnell erkannt und umfangreich gehandelt, um die deutschen Kommunen auskömmlich zu finanzieren. In Summe sind die Kommunen dank dieser Hilfen sehr gut durch die beiden Krisenjahre gekommen.

1 Siehe Gesetz zur finanziellen Entlastung der Kommunen und der neuen Länder vom 6. Oktober 2020, BGBl. I S. 2072.

**Prof. Ronny Freier, Ph. D.**, ist Professor für Öffentliche Finanzwirtschaft und Haushaltsrecht an der Technischen Hochschule Wildau.

## Überschüsse in den Jahren 2020 und 2021

Um die gesamtheitliche Lage der kommunalen Finanzen eines Jahres zu bewerten, wird zumeist der kommunale Finanzierungssaldo herangezogen. Abbildung 1 zeigt die Entwicklung des Finanzierungssaldos von 2017 bis 2021. In den Jahren vor der Krise (2017-2019) konnten die Kommunen ihre Haushalte mit enormen Überschüssen abschließen. In nur drei Jahren wurden fast 25 Mrd. Euro an Überschüssen gebildet. Und auch in den Haushaltsplänen für 2020 und 2021 waren weitere Einnahme- und Ausgabesteigerungen eingeplant.

Die Coronakrise traf die Kommunen im März 2020 unvermittelt. Auf der Ausgabenseite mussten Kommunen die aktuelle Krisenbewältigung finanzieren. Dazu gehörten die Organisation in den Gesundheitsämtern, die Sicherung der Daseinsvorsorge und des Schulbetriebs, die Erstattung von Kitabeiträgen und die coronabedingten Mehraufwendungen in kommunalen Krankenhäusern. Auch die Kosten für Sozialleistungen waren im Frühjahr/Sommer 2020 bereits deutlich erhöht (Boettcher et al., 2021a, Kapitel F, 17). Die tatsächlichen Mehraufwendungen der Kommunen durch die Coronakrise sind bis heute schwer zu ermitteln und wurden in vielen Bereichen direkt durch Unterstützung von Ländern und Bund abgedeckt. Bedeutsamer als die Mehraufwendungen waren allerdings die enormen Steuereinbußen auf der Einnahmeseite. Hier sind insbesondere die gravierenden Rückgänge 2020 bei der extrem konjunkturabhängigen Gewerbesteuer zu nennen. Insgesamt wurden 2020 10,3 Mrd. Euro weniger bei der Gewerbesteuer (brutto) vereinnahmt als 2019 (7,7 Mrd. Euro in den Flächenländern). Aber auch beim kommunalen

Abbildung 1

**Kommunales Finanzierungssaldo**

in Mrd. Euro

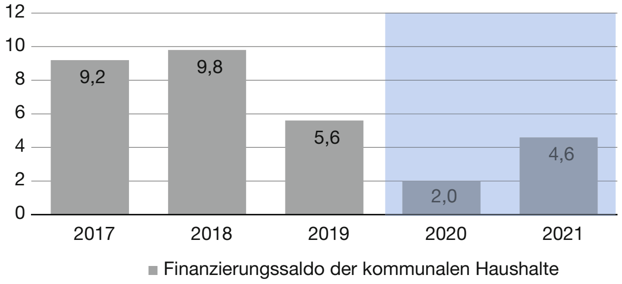

Quelle: eigene Darstellung auf Basis der Rechnungsstatistik 2017, 2018 (FS 14-2) und der Kassenstatistik 2019-2021 (FS 14-3.3).

Abbildung 2

**Entwicklung bei den Gewerbesteuereinnahmen**

in Mrd. Euro, quartalsweise

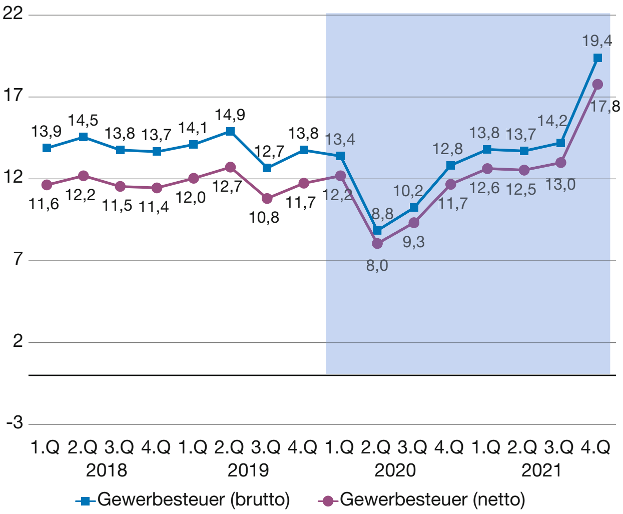

Quelle: eigene Darstellung auf Basis der Steuerstatistik 2016-2021 (FS 14-4).

Anteil an der Einkommensteuer waren Rückgänge zu beobachten (1,6 Mrd. Euro). Angesichts der steigenden Aufwendungen und enormer Einbrüche bei den Steuern, ist die Entwicklung mit positiven Haushaltsabschlüssen von +2 Mrd. Euro in 2020 und +4,6 Mrd. Euro in 2021 sehr bemerkenswert (vgl. Abbildung 1). Die Kommunen konnten die Krisenjahre trotz der genannten Herausforderungen demnach positiv bewältigen. Die Gründe für diese Entwicklungen werden im Folgenden erläutert.

## Enorme Steuereinbußen 2020, starkes Plus 2021

Um die Entwicklung der kommunalen Finanzen 2020 und 2021 zu verstehen, sind die Einnahmen aus der Gewerbesteuer der zentrale Ausgangspunkt. Abbildung 2 zeigt die quartalsweise Entwicklung der Gewerbesteuereinnahmen von 2018 bis 2021. In den Vorkrisenjahren wurden bei der Gewerbesteuer – der wichtigsten Einnahmequelle der Kommunen – Rekordwerte vermeldet. 2019 wurden von den Kommunen in den Flächenländern 50,34 Mrd. Euro bei der Gewerbesteuer (brutto) vereinnahmt. Nach Zahlung der Gewerbesteuerumlage an Bund und Länder verblieben davon 42,63 Mrd. Euro netto in den kommunalen Haushalten.

Das Coronajahr 2020 begann im ersten Quartal mit Gewerbesteuerzahlungen auf vergleichbarem Niveau. Der große Einbruch begann aber schon im zweiten Quartal 2020. Hier wurden lediglich 8,8 Mrd. Euro an Gewerbesteuern (brutto) eingenommen, ein Rückgang von mehr als 40 % gegenüber dem Vorjahresquartal. Ausschlaggebend für die sofortigen Auswirkungen bei den Gewerbesteuerzahlungen war der Beschluss des Bundesfinanzministeriums, dass Unternehmen ihre vierteljährlichen Abschlagszahlungen der Gewerbesteuer aufgrund von eingetrübten Gewinnerwartungen unbürokratisch bereits im zweiten Quartal mindern konnten. Das dritte und vierte Quartal 2020 wurden ebenfalls mit schlechteren Werten als im Vorjahr abgeschlossen. Insgesamt deutete sich aber eine leichte Erholung an. Im vierten Quartal lagen die Einnahmen bei der Gewerbesteuer (brutto) nur noch knapp 1 Mrd. Euro unter dem Vorjahresquartal (ein Rückgang um 7 %). Fachleute hatten die massiven Einbrüche bei der Gewerbesteuer im zweiten Quartal 2020 vorausgesehen (Freier und Geißler, 2020), und auch die zum Teil düsteren Prognosen der Wirtschaftsforschungsinstitute (ifo, 2020; SVR, 2020) hatten die Bundes- und Landespolitik frühzeitig alarmiert. Schon im Mai wurde klar, dass Bund und Länder die Kommunen umfangreich stützen werden. Am 3. Juni wurde durch die Bundesregierung der „kommunale Solidarpakt“ beschlossen, in dem unter anderem die Ausfälle bei der Gewerbesteuer kompensiert werden sollten. Das entsprechende Gesetz zur finanziellen Entlastung der Kommunen wurde am 17. September 2020 vom Bundestag beschlossen.

Die Ausgleichszahlungen für Gewerbesteuermindereinnahmen der Kommunen stellen die wichtigste Einzelmaßnahme bei der Unterstützung der Kommunen durch Bund und Länder dar. Bei dieser Maßnahme wurden 11,46 Mrd. Euro (davon 10,84 Mrd. Euro an die Kommunen in den Flächenländern) verteilt (Boettcher et al., 2021a, Kapitel E, 11). Die Kosten für diese Hilfen wurden hälftig durch Bund und Länder getragen. Abbildung 3 veranschaulicht den Effekt dieser finanziellen Hilfe für die Kommunen in den Flächenländern. Verglichen werden die Zahlungen der Gewerbesteuer von 2019 bis 2021 und der Ausgleichszahlung 2020. Wurden 2019 50,34 Mrd. Euro an Gewerbesteuereinnahmen (brutto) vereinnahmt, so addierten sich die Einnahmen 2020 mit 41,39 Mrd. Gewerbesteuer plus 10,84 Mrd. Euro Ausgleichszahlungen auf 52,23 Mrd.

Euro. Die Tatsache, dass das Gewerbesteueraufkommen 2020 unter Einbeziehung der Ausgleichszahlungen sogar leicht über dem Niveau von 2019 gelegen hat, erklärt sich durch die pauschale Übernahme von Ausfällen durch den Bund und die Länder. Um ein vereinfachtes und zügiges Verfahren zu gewährleisten, wurden die Hilfen im Gesetz pauschaliert. Es wurden also nicht die exakten Ausfälle ermittelt, sondern die Zahlungen basierten auf den geschätzten Abweichungen (als Basis dienten die Zahlen aus den Steuerschätzungen vom Oktober 2019 und Mai 2020). Das Jahr 2021 stellt sich bei den Gewerbesteuereinnahmen gänzlich anders dar. Trotz der anhaltenden Coronapandemie und der Tatsache, dass weiterhin Coronamaßnahmen die Wirtschaft belasteten, konnten 2021 Rekordwerte bei den Gewerbesteuern erwirtschaftet werden. Insgesamt wurden 2021 Gewerbesteuern (brutto) in Höhe von 55,4 Mrd. Euro in den Flächenländern erwirtschaftet. Damit lagen die Bruttoeinnahmen um 33,9 % über den Einnahmen von 2020 (ohne Ausgleichszahlung) und sogar 10,1 % über den Einnahmen von 2019.

Abbildung 2 zeigt auch den interessanten quartalsweisen Verlauf im Jahr 2021. Die ersten drei Quartale 2021 waren in der Höhe der Einnahmen durchweg vergleichbar mit dem Einnahmeniveau von 2019. Das 4. Quartal 2021 aber stellt eine Ausnahme dar; es ist das Quartal mit den historisch höchsten jemals gemessenen Einnahmen. Wurden im 3. Quartal 2021 noch 14,2 Mrd. Euro bei der Gewerbesteuer (brutto) vereinnahmt, so waren es im 4. Quartal plötzlich 19,4 Mrd. Euro – ein Anstieg um 36,5 %. Zu vermuten ist, dass dieser Sondereffekt durch eine spezielle Situation bei den Abschlagszahlungen der Unternehmen begründet ist. Nachdem die Unternehmensgewinne (und damit auch die Gewerbesteuer) 2020 krisenbedingt sehr niedrig waren, mussten die Unternehmen auf der Grundlage dieser Gewinne im 1. bis 3. Quartal 2021 auch verhältnismäßig niedrige Quartalsabschläge zahlen. Erst zum Ende des Jahres wurden die tatsächlich sehr hohen Gewinne des Jahres 2021 vollständig in die Gewerbesteuerzahlung transferiert.

Neben den Ausgleichszahlungen für Gewerbesteuermindereinnahmen 2020 gab es einen weiteren Effekt bei der Gewerbesteuer, der die finanzielle Lage der Kommunen im Krisenjahr 2020 beeinflusste. Zum 1.1.2020 trat eine schon im Vorfeld beschlossene Änderung bei der Gewerbesteuerumlage in Kraft. Die Gewerbesteuerumlage wird seit 1969 erhoben. Die Kommunen müssen ihre Gewerbesteuereinnahmen dadurch mit Bund und Ländern teilen (BMF, 2022). Die genauen Regelungen und Sätze haben sich über die Jahre immer wieder verändert. Von besonderem Interesse war eine Erhöhung der Gewerbesteuerumlage im Jahr 1995. Hier wurde die Umlage in den westdeutschen Ländern erhöht, um die westdeutschen Kommunen an den Kosten der Finanzierung des Solidarpakts

Abbildung 3
**Einnahmen aus der Gewerbesteuer und Ausgleichszahlungen 2020 in den Flächenländern**
in Mrd. Euro

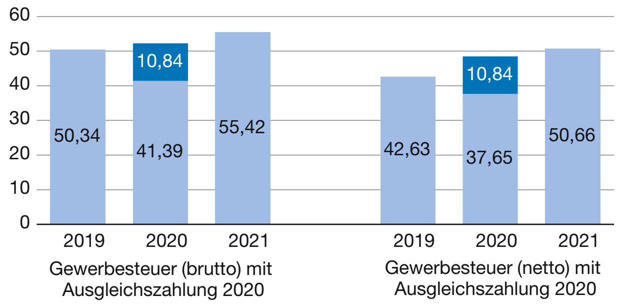

Quelle: eigene Darstellung auf Basis der Steuerstatistik 2019-2021 (FS 14-4)

zu beteiligen. Diese Erhöhung wurde zum 1.1.2020 mit dem Auslaufen des Solidarpakts gestrichen. Im Ergebnis steht für die (westdeutschen) Kommunen eine geringere Zahlung der Gewerbesteuerumlage.

Die Abbildungen 2 und 3 zeigen den daraus resultierenden Effekt. In Abbildung 2 sind die Einnahmen der Gewerbesteuer netto (Bruttoeinnahmen minus Gewerbesteuerumlage) als zusätzliche Linie unter den Bruttoerträgen eingezeichnet. Deutlich zu erkennen ist, dass diese Linie sich ab 2020 im Abstand an die Bruttoerträge angenähert hat. Noch deutlicher wird der Gesamteffekt in Abbildung 3. 2019 mussten die Kommunen 7,71 Mrd. Euro der Bruttoeinnahmen bei der Gewerbesteuer über die Gewerbesteuerumlage mit Bund und Ländern teilen. Dies entspricht einem Anteil von 15,3 %. 2020 sank die Gewerbesteuerumlage (bei niedrigeren Einnahmen) auf 3,7 Mrd. Euro und machte nur noch einen Anteil von 9 % aus. Die veränderten Regelungen bei der Gewerbesteuerumlage trugen 2020 demnach mit 2,7 Mrd. Euro zu einem positiven Finanzergebnis der Kommunen bei. Und dieser Effekt ist – anders als bei den Ausgleichszahlungen – von dauerhafter Natur. Auch 2021 konnten die Kommunen von der verringerten Gewerbesteuerumlage profitieren. 2021 macht dieser Einspareffekt am Ende etwa 3,6 Mrd. Euro aus. Damit hilft die Absenkung der Gewerbesteuerumlage den (westdeutschen) Kommunen kontinuierlich und trug 2021 entscheidend zu dem positiven Gesamtergebnis bei.

## Kostenübernahme bei den KdU hilft den Kommunen

Die Entwicklung der Steuereinnahmen bei der Gewerbesteuer und die Maßnahmen der Ausgleichszahlungen sowie die Reform der Gewerbesteuerumlage waren maßgeblich für die Entwicklung der kommunalen Finanzen 2020 und 2021. Zusätzlich gab es aber eine weitere Ver-

änderung bei den kommunalen Finanzen, die die Jahresergebnisse der kommunalen Haushalte entscheidend mit beeinflusste. Der Bund übernimmt seit der Einführung der Kosten für Unterbringung und Heizung (KdU) 2005 einen Anteil der entstandenen Kosten.[2] Weil die Kommunen bei dieser Sozialaufgabe kaum Gestaltungsspielraum haben und die Kosten insbesondere Kommunen mit vielen finanziell schwachen Haushalten überproportional belasten, wurde von Wissenschaft und Praxis schon lange eine Erhöhung des Anteils des Bundes gefordert (Arnold et al., 2015 sowie Geissler und Niemann, 2015). Der Bund hatte sich einer erhöhten Übernahme lange verweigert, auch weil eine Erhöhung des Anteils auf über 50 % grundgesetzliche Probleme mit sich brachte. Als Reaktion auf die Coronapandemie und die möglichen Konsequenzen für die kommunalen Haushalte wurde die Bundesbeteiligung an den KdU dauerhaft um 25 Prozentpunkte erhöht.[3]

Der Entlastungseffekt dieser Maßnahme ist enorm und führt zu direkter Kostensenkung insbesondere in Kommunen mit schwacher Sozialstruktur (und damit höheren finanziellen Problemen). 2020 lagen die KdU bei insgesamt 14,4 Mrd. Euro (11,9 Mrd. Euro in den Flächenländern). In 2021 waren die KdU gleichbleibend hoch mit ebenfalls 14,4 Mrd. Euro bundesweiten Ausgaben (12 Mrd. Euro in den Flächenländern). Der Entlastungseffekt durch die zusätzliche Übernahme von 25 % wird in Tabelle 1 für alle Flächenländer dargestellt. Da die Reform die Bundesbeteiligung dauerhaft verändert, wurden die Haushalte 2020 und 2021 entlastet. Die Kommunen in NRW wurden z. B. durch die Erhöhung der Bundesbeteiligung an den KdU 2020 und 2021 um jeweils mehr als 1 Mrd. Euro entlastet. Auch die ostdeutschen Flächenländer konnten enorm profitieren, mit einer Entlastung von 466 Mio. Euro 2020 und 453 Mio. Euro 2021. Insgesamt lag die Entlastungswirkung durch die KdU-Reform in beiden Jahren bei knapp unter 3 Mrd. Euro (in den Flächenländern).

## Positivtrend bei den Kassenkrediten hält an, Investitionen auf Rekordniveau

Die Tatsache, dass die kommunalen Haushalte die Jahre 2020 und 2021 mit Überschüssen abschließen konnten, zeigt auch Auswirkungen auf andere Finanzindikatoren. Abbildung 4 zeigt die vierteljährliche Entwicklung der Kassenkredite vor und während der Coronajahre. Deutlich wird, dass der positive Abwärtstrend auch in den Krisenjahren beibehalten werden konnte. Zwar stieg der Liquiditätsbedarf zum Ende des 1. Quartals 2020 kurzfristig an, die Kassenkredite schlossen 2020 aber – dank der Ausgleichszahlungen für Gewerbesteuerausfälle – im 4. Quartal 2020 positiv ab. Auch 2021 setzte sich dieser Positivtrend weiter fort. Am Ende des Jahres 2021 lagen die Kassenkredite der Kommunen in den Flächenländern erstmals wieder bei unter 30 Mrd. Euro.

Von besonderer Bedeutung sind zudem die kommunalen Investitionen (hier Sachinvestitionen der kommunalen Haushalte). Zu Beginn der Coronakrise stand zu befürchten, dass die kurzfristigen Steuerausfälle zu Haushaltsschieflagen in einem bedeutenden Anteil der deutschen Kommunen führen könnten. Im Ergebnis drohten weitreichende Haushaltssperren und insbesondere Kürzungen bei den geplanten Investitionen. Es ist den umfangreichen Maßnahmen von Bund und Ländern geschuldet, dass diese Reaktionskette in der Krise nicht eingetroffen ist. Wie Abbildung 5 zeigt, konnten die Kommunen ihre Investitionen wie geplant durchführen. Sowohl 2020 als auch 2021 wurden Investitionen in Höhe von 38,6 Mrd. Euro getätigt. Diese Investitionen stellen dringend benötigte Infrastruktur für die Zukunftsfähigkeit Deutschlands dar[4] und sie leisten einen wichtigen Beitrag zur Stabilisierung der Wirtschaft in der Krise.

## Die Herausforderungen für die kommunalen Finanzen liegen in der Zukunft

Während die beiden vergangenen Krisenjahre für die kommunalen Haushalte demnach gut gemeistert wurden, liegen die Herausforderungen für die kommunalen Finanzen in der Zukunft. Die Coronapandemie ist noch nicht beendet und wird die Kommunen voraussichtlich auch in den kommenden (Winter-)Monaten beschäftigen. Dazu kommen die Belastungen durch den Krieg in der Ukraine und die hiermit verbundenen politischen und wirtschaftlichen Folgen. Tatsächlich stehen bei den Kommunen im Moment die (wirtschaftlichen) Folgen des russischen Angriffs auf die Ukraine im Mittelpunkt. Die Kommunen sind direkt von den gestiegenen Energiepreisen und der drohenden Energieknappheit betroffen. Zudem sind viele Unternehmen im Energiesektor teilweise oder gänzlich in kommunaler Trägerschaft. Die mit den gestiegenen Energiepreisen und einer drohenden Energieknappheit verbundenen Risiken für das Wirtschaftswachstum erschweren zudem die Planungssicherheit in den Haushalten.

Getrieben durch die Energiekrise – aber auch aufgrund von Problemen in den Lieferketten – belastet auch die In-

2 Für eine detaillierte Darstellung der Entwicklung der verschiedenen Anteile siehe Wixforth (2016) sowie Boettcher et al. (2021b).

3 Siehe Gesetz zur finanziellen Entlastung der Kommunen und der neuen Länder vom 6. Oktober 2020, BGBl. I S. 2072.

4 Das KfW Kommunalpanel stellt auch 2022 wieder fest, dass der kommunale Investitionsbedarf enorm ist und 2022 trotz des hohen Investitionsvolumens weiterhin gewachsen ist. Der Bedarf wird 2022 mit 159,4 Mrd. Euro beziffert (KfW, 2022).

Tabelle 1

**KdU in den Flächenländern und Entlastungseffekt durch die zusätzliche Übernahme des Bundes**

in Mio. Euro

| | Kosten KdU 2020 | Entlastung 2020 | Kosten KdU 2021 | Entlastung 2021 |
|---|---|---|---|---|
| Schleswig-Holstein | 525 | 131 | 524 | 131 |
| Niedersachsen | 1.330 | 332 | 1.329 | 332 |
| Nordrhein-Westfalen | 4.106 | 1.026 | 4.131 | 1.033 |
| Hessen | 1.103 | 276 | 1.117 | 279 |
| Rheinland-Pfalz | 515 | 129 | 519 | 130 |
| Baden-Württemberg | 1.196 | 299 | 1.238 | 309 |
| Bayern | 1.100 | 275 | 1.130 | 282 |
| Saarland | 206 | 51 | 204 | 51 |
| Brandenburg | 373 | 93 | 362 | 91 |
| Mecklenburg-Vorpommern | 270 | 67 | 264 | 66 |
| Sachsen | 558 | 140 | 546 | 137 |
| Sachsen-Anhalt | 405 | 101 | 387 | 97 |
| Thüringen | 257 | 64 | 247 | 62 |
| | | | | |
| Flächenländer Ost | 1.863 | 466 | 1.805 | 451 |
| Flächenländer West | 10.079 | 2.520 | 10.191 | 2.548 |
| Alle Flächenländer | 11.942 | 2.986 | 11.996 | 2.999 |

Quelle: eigene Darstellung auf Basis der Daten der Bundesagentur für Arbeit zu den Kosten der Unterbringung und Heizung.

Abbildung 4

**Entwicklung der Kassenkredite**

in Mrd. Euro, quartalsweise 2018-2021

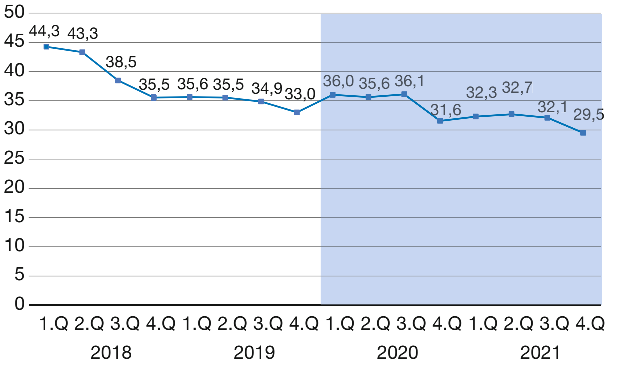

Quelle: eigene Darstellung auf Basis der vorläufigen Schuldenstatistik 2018-2021 (FS 14-5.2).

flation die Kommunen in der Zukunft. Zwar werden mit der Inflation – kurzfristig – auch die Steuereinnahmen steigen (dies gilt insbesondere für den Gemeindeanteil an der Umsatzsteuer), aber die Kommunen sehen sich schon jetzt in vielen Bereichen mit höheren Preisen konfrontiert. Insbesondere im Bausektor steigen die Rohstoffpreise überproportional und machen die kommunalen Investitionen teurer. Die zunächst bestehende Sorge zahlreicher Kommunen, durch die hohe Zahl an ukrainischen Flüchtlingen finanziell stark belastet zu werden, wurde durch die Vereinbarung zwischen dem Bund und den Ländern vom 7. April 2022 abgeräumt. Demnach erhalten Geflüchtete aus der Ukraine ab dem 1. Juni 2022 Leistungen nach dem SGB II beziehungsweise SGB XII. Zudem hat der Bund zugesagt, die Länder und Kommunen für 2022 über einen erhöhten Anteil der Länder an der Umsatzsteuer zunächst mit insgesamt 2 Mrd. Euro bei den Mehraufwendungen für die Unterbringung und Betreuung zu unterstützen. Ergänzend ist darauf hinzuweisen, dass der Bund ohnehin den Großteil der im Rechtskreis SGB II anfallenden Kosten trägt.

Bei den Steuereinnahmen ist 2022 trotz der pessimistischen Ausblicke bislang kein Einbruch bei den Kommunen zu sehen. Auch durch die angesprochene hohe Inflation liegen die Einnahmen im ersten Quartal 2022 deutlich über den Einnahmen im Vorjahresquartal. Auch wenn eine genaue Prognose bei allen Widrigkeiten schwerfällt, so soll dieser Trend gemäß den Ergebnissen der Mai-Steuerschätzung auch in den kommenden Jahren anhalten. Demnach werden die Kommunen in den Jahren 2022 bis 2024 fast 10 Mrd. Euro mehr Aufkommen erzielen als in der letzten Steuerschätzung vor dem Beginn der Coronapandemie (156. Sitzung des Arbeitskreises „Steuerschätzungen" vom Oktober 2019) für den Zeitraum 2022 bis 2024 prognostiziert wurde. Dies ist insbesondere deshalb bemerkenswert, da die Steuerschätzung im November 2020 davon ausging, dass sich für diesen Zeitraum eine Aufkommensverschlechterung (gegenüber der Steuerschätzung vom Oktober 2019) um nahezu 28 Mrd. Euro ergibt. Im Hinblick auf die Steuerentwicklung und -prognose konnte die Krise demnach in geradezu atemberaubender Geschwindigkeit überwunden werden.

Ein weiteres Thema sind die Zinsen. Hier ist eine Wende mittlerweile nicht nur im Bereich des Möglichen, sondern hoch wahrscheinlich. In den vergangenen Wochen sind vor allem die langfristigen Zinsen wieder deutlich gestiegen (die Umlaufsrendite der Bundeswertpapiere mit langer Laufzeit ist um 1 Prozentpunkt nach oben geklettert, nachdem sie seit Anfang 2019 im negativen Bereich lag). Hier zahlt es sich jetzt aus, dass insbesondere die kommunalen Kassenkredite in den vergangenen Jahren deutlich zurückgefahren werden konnten und die Kommunen damit weniger stark direkt von Zinsänderungen betroffen sind. Allerdings gibt es bei den Kassenkrediten eine starke Ungleichverteilung, sodass einige Kommunen mit hohen Kassenkreditbeständen durchaus eine spürbare Mehrbelastung durch Zinsaufwendungen ver-

Abbildung 5

**Sachinvestitionen in den kommunalen Haushalten**

in Mrd. Euro

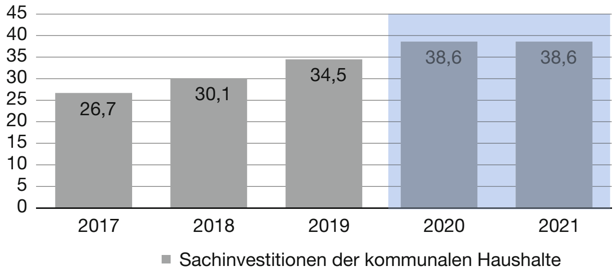

Quelle: eigene Darstellung auf Basis der Kassenstatistik 2017-2021 (FS 14-2).

zeichnen könnten. Da die kommunalen Investitionskredite meist sehr langfristig abgeschlossen wurden, bestehen hier im Bestand auch eher geringe kurzfristige Risiken für die kommunalen Haushalte. Klar ist aber auch, dass die höheren Zinsen für die aktuelle Investitionstätigkeit der Kommunen ein Problem darstellen.

## Fazit

Die deutschen Kommunen sind in fiskalischer Hinsicht bemerkenswert gut durch die Krisenjahre 2020 und 2021 gekommen: Trotz der pandemiebedingten Belastungen konnten die Kommunen in beiden Jahren Finanzierungsüberschüsse erzielen, den Abbau der Kassenkredite fortsetzen und die Investitionstätigkeit weiter stärken. Die Ergebnisse der Jahre 2020 und 2021 unterscheiden sich damit maßgeblich von den fiskalischen Entwicklungen im Zuge der Banken- und Wirtschaftskrise etwas mehr als zehn Jahre zuvor; insbesondere 2009 und 2010 haben die Kommunen mit hohen Defiziten abgeschlossen, die zu einem starken Anstieg der Kassenkreditschulden geführt haben.

Das insgesamt positive fiskalische Abschneiden der Kommunen ist im Wesentlichen das Resultat umfassender finanzieller Hilfen von Bund und Ländern, die hiermit die Handlungsfähigkeit der Kommunen in der Pandemie sicherstellen und eine erneute Schuldenkrise bei den Kommunen verhindern konnten. Die positive Entwicklung 2021 ist neben anhaltenden finanziellen Unterstützungsleistungen von Bund und Ländern vor allem auf das Wiederanspringen der kommunalen (Gewerbe-) Steuereinnahmen zurückzuführen. Laut den Ergebnissen der aktuellen Steuerschätzung soll es zudem bei einem nachhaltigen Steuerwachstum für die kommunalen Steuern bleiben. Die deutlich verbesserte Perspektive bei den Steuereinnahmen darf jedoch nicht darüber hinwegtäuschen, dass auch die finanzielle Entwicklung der Kommunen in Anbetracht der aktuell bestehenden politischen und wirtschaftlichen Risiken (vor allem infolge des russischen Angriffs auf die Ukraine) durch ein überaus hohes Maß an Unsicherheit und Ungewissheit geprägt ist. Ob die Erholung weiter anhält, bleibt daher abzuwarten.
